# Isolation and identification of *Mannheimia haemolytica* by culture and polymerase chain reaction from sheep’s pulmonary samples in Shiraz, Iran

**DOI:** 10.14202/vetworld.2018.636-641

**Published:** 2018-05-17

**Authors:** Mohammad Tabatabaei, and Fatemeh Abdollahi

**Affiliations:** Department of Pathobiology, School of Veterinary Medicine, Shiraz University, Shiraz, Iran

**Keywords:** *Mannheimia haemolytica*, pneumonia, polymerase chain reaction, sheep, Shiraz

## Abstract

**Background and Aim:**

*Mannheimia haemolytica* is a Gram-negative, non-motile, and non-spore-forming rod-shaped coccobacillus bacterium. On blood agar plate, it shows complete hemolysis. This bacterium constitutes a part of normal flora of the upper respiratory system of ruminants. It is considered as the opportunistic pathogen and the main factor of pneumonic pasteurellosis, which has caused a severe economic loss in sheep and cattle industries. Considering the prevalence of the disease in sheep and goat population in the dry and hot regions of the country in general and in Fars province in particular in the form of pneumonia, the purpose of this study was to isolate and identify the bacterium *M. haemolytica* from the lung tissues of sheep slaughtered in Shiraz abattoir through culturing and polymerase chain reaction (PCR) methods.

**Materials and Methods:**

In this study, a total of 2500 sheep’s lungs were evaluated for finding pneumonic effects. Then, 161 infected pneumonic samples of lung tissues were investigated by culture and PCR methods.

**Results:**

After cultivation, purification, and DNA extraction, 38 samples were found positive for *M. haemolytica* by cultivation and then all the 38 isolates were confirmed by PCR and multiplex PCR (mPCR). Results of this study indicated that culture and PCR are both practical in identification and isolation of this bacterium though culture is more time-consuming. The utilized mPCR has been more successful in the identification of the bacteria since it requires less time and cost.

**Conclusion:**

In this study, PCR as a superior method among other methods of bacteriology for fast examination of infectious diseases and mPCR, which is a valuable tool for identification of *M*. *haemolytica* in clinical samples of animals, was used.

## Introduction

*Mannheimia haemolytica* is a Gram-negative, non-motile, non-spore-forming, and rod-shaped coccobacillus bacterium. The G+C content of the DNA is about 39-44% [1]. In 1999, Angen *et al*. [2] reclassified the A biotypes of *Pasteurella haemolytica* into a new genus named *M. haemolytica* after the name of German biologist Walter Mannheimia. He improved the study and classification of *Pasteurellaceae* family through his investigations [3].

*M. haemolytica* is found as a natural flora on the mucous membrane of the upper respiratory tract of ruminants. Most of its known species were isolated from cattle, sheep, and goats. Virulence factors of *M. haemolytica* include adhesins, capsule, lipopolysaccharide, outer membrane proteins, iron-regulated proteins, and leukotoxin. The main pathogen of bovine pneumonic pasteurellosis (shipping fever) is *M. haemolytica* which can be the causative agent of pasteurellosis in sheep and goats. In addition, it causes two syndromes of pneumonia and septicemia in sheep and goats, respectively, and leads to gangrenous mastitis in ewes [4].

Therefore, the purpose of the current study was to identify and isolate *M. haemolytica* from slaughtered sheep’s lungs in Shiraz abattoirs through culture and polymerase chain reaction (PCR). Considering the significant role of this bacterium in sheep pneumonia, the effectiveness of these two methods in bacterial identification with improved sensitivity and rapidity was investigated.

## Materials and Methods

### Ethical approval

Ethical approval was not necessary as samples were collected from dead animals.

### Sample collection

From October 2015 to June 2016, following the slaughter of the apparently healthy animals, all the lungs (≈2500) were inspected according to the standard postmortem meat inspection procedure; and then, 161 lungs that contain hepatization and consolidation in cranioventral lobes were selected for further examination. Each lung sample was separately placed in sterile zip-lock plastic bags and transferred to the laboratory of Bacteriology, Faculty of Veterinary Medicine, Shiraz University, in about 2 h, in cool condition. Samples were collected from cranioventral lungs’ lobes with hyperemia, rough surface, and consolidation symptoms.

### Culture and isolation

Upon inspection, the surface of each suspected lung was sterilized with a hot iron blade and incised using sterile scalpel blade and the inner surface of the incision was sampled with a sterile swab. Following that, the swabs were streaked on blood agar plates and incubated aerobically at 37°C for 24-48 h.

Then, for isolation, a loopful of the identified colonies were streaked over a new blood agar plate and incubated aerobically at 37°C for 24 h. Next, from culture-positive plates, typical colonies were subjected to Gram’s staining to study staining reactions and cellular morphology under a light microscope. Mixed and Gram-negative bacteria were further subcultured with due care, on both blood and MacConkey agar plates for final identification.

The growth of typical colonies on both blood agar and MacConkey agar plates was characterized using blood agar plate for the presence of hemolysis, the type of hemolysis, the general appearance of colonies (morphology, color, shape, size, and consistency) and MacConkey agar plates for the ability to ferment lactose. Pure cultures of single colony type were further analyzed by catalase and oxidase tests. Final identification of bacteria to species level was aided using the biochemical tests including Metabolism of sugars such as glucose, sorbitol, lactose, sucrose, maltose, and mannose and tests for metabolic end products such as indole (Peptone water, Merck Co., Germany) following standard procedures. If the organism can produce a narrow zone of hemolysis on blood agar plate and grown on MacConkey agar plate, but unable to produce indole, it is interpreted as *M. haemolytica* [4].

### DNA extraction

A few colonies from the phenotypically characterized pure cultures of *M. haemolytica* from 24-48 h growth on blood agar plates were transferred into 1.5-ml Eppendorf tubes. The bacterial genomic DNA was extracted according to Gram-negative DNA extraction kit (Cinnagen, Iran). The extracted DNA was determined to be of good quality according to A260/A280, and DNA concentration was measured using Nanodrop (10000 V 3.52). Finally, the extracted DNA was stored at −20°C until use.

### PCR

The oligonucleotide primers used in this study are listed in Table-1 [5]. The reaction mixture solution for PCR was prepared using 2.5 μl of 10× PCR buffer, 1.5 μl (1.5 mM) MgCl**_2_**, 1.5 μl (200 mM) dNTPs, 1.5 µl (100 nmol) each primer of *M. haemolytica*, 0.3 U of *Taq* DNA polymerase, 13.2 μl H_2_O, and 3 μl of DNA template to have a final volume of 25 μl. The master mix form mPCR is also shown in Table-2. PCR conditions were optimized by putting gradient PCRs with annealing temperature ranging from 50.5°C to 50.7°C using 0.2-ml thin-walled PCR tubes. After initial denaturation (at 94°C for 4 min), amplification conditions were as follows: Denaturation at 94°C for 40 s, annealing at 50.5-50.7°C for 40 s, and extension at 72°C for 40 s. This was repeated for 30 cycles in a Block assembly 96G thermocycler with a hot top assembly (Analytic Jena, Germany), with a final extension of 72°C for 6 min, and the PCR products remained in the thermal cycler at 4°C until they were collected (M. Tabatabaei, unpublished data). The preparation of the reagents for PCR, the addition of the DNA extracts to the mixture, and the PCR thermocycling was performed in three separate rooms to prevent crossover contamination by extraneous nucleic acids.

**Table-1:** The utilized PCR primers.

Primer name	Primer sequence 5′ to 3′	Amplicon Size (bp)	References
Lkt	(F) GCAGGAGGTGATTATTAAAGTGG	206	[5]
	(R) CAGCAGTTATTGTCATACCTGAAC		
HP	(F) CGAGCAAGCACAATTACATTATGC	90	[5]
	(R) CACCGTCAAATTCCTGTGGATAAC		
16S	(F) GCTAACTCCGTGCCAGCAG	304	[5]
	(R) CGTGGACTACCAGGGTATCTAATC		

**Table-2:** The amount and characteristics of mPCR components.

The utilized components	DNA Template	*Taq DNA* polymerase	Reverse primer (Each)	Forward primer (Each)	dNTPs	MgCl_2_	PCR Buffer	H_2_O
Density	-	5 U/µl	20 µM	20 µM	10 mM	50 mM	10×	-
Amount (ml)	2	0.3	1.5	1.5	2	1.8	2.5	7.4

A negative control consisting of all components of reaction mixture except the DNA template was included in all PCR experiments. Positive controls were included in the PCR from colonies that were confirmed according to NCBI gene bank sequencing. After initial identification of isolates according to phenotypic characteristics and biochemical tests, some isolates and *M. hemolytica* from the bacterial collection of the Faculty of Veterinary Medicine, Shiraz University, were sent for sequencing of 16s DNA-PCR products, and then these bacterial strains were used as positive control in PCR.

To evaluate the PCR results, electrophoresis in agarose gel was carried out. PCR amplicons were assessed by loading 8 µl of the PCR product plus 2 µl of loading buffer into separate wells of 2% (w/v) agarose gel (Agarose I; Cinnagen, Iran) containing ethidium bromide (0.5 µg/ml). A molecular weight marker (50 bp; Cinnagen, Iran) was loaded into the first well to determine the size of the amplified fragments. The gel was immersed in TBE buffer and subjected to a voltage difference of 100 V that led to the separation of the fragments. Visualization was undertaken using an ultraviolet transilluminator (BTS-20, Japan), and the resulting image was captured by a computer software program (AlphaEase; Alpha Innotech, Genetic Technologies, Inc. Miami).

For the final confirmation of isolates of *M. haemolytica*, PCR products of some samples according to the Macrogene Company’s instructions were prepared at 50 μl volume and were transferred for sequencing. Sequence analysis was performed on an ABIPrism 3100 Genetic Analyzer (Applied Biosystems, Inc. Thermo Fisher Scientific Corporation. USA).

## Results

**Figure-1:**
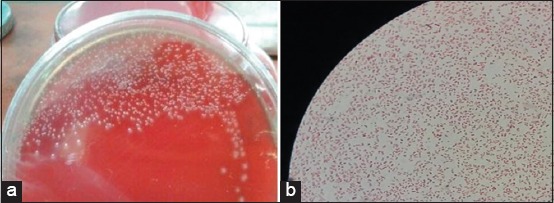
*M. haemolytica* colonies on (a) blood agar plate and (b) Gram staining smear.

Identification of the bacterial species was made by observation of their colonial morphology, Gram staining reaction, and biochemical characteristics [4]. Through culture, 38 isolates of *M. haemolytica* with small gray circle colonies with β-hemolysis were observed that were rod-shaped in Gram staining (Figure-1). Catalase and oxidase tests were positive, but indole test was negative. The highest level of isolation was observed in January, February, and March.

In addition, among the cultured tissues of lungs studied in this research, other bacteria such as *Staphylococcus, Streptococcus, Corynebacterium, Micrococcus, Rhodococcus, Acinetobacter, Bacillus*, and *Pasteurella* were identified and isolated. These bacterial isolates were identified according to phenotypic characteristics and biochemical tests.

**Figure-2:**
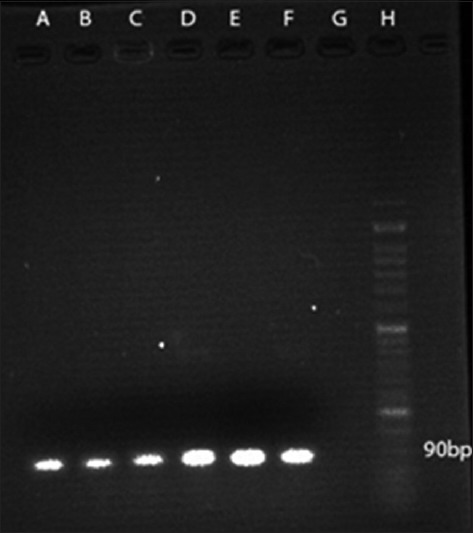
PCR amplification profile of *M. haemolytica* from DNA isolated directly from samples with HP primer. Lanes A to E: Positive samples (90 bp amplicon size), Lane F: Positive control, Lane G: Negative control, Lane H: 50 bp DNA marker.

**Figure-3:**
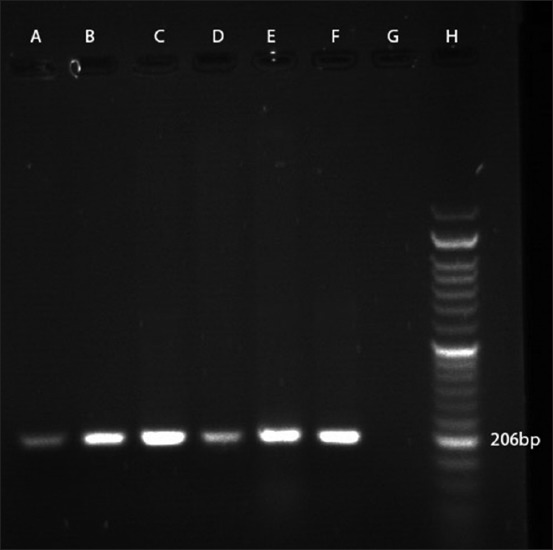
PCR amplification profile of *M. haemolytica* from DNA isolated directly from samples with LKT primer. Lanes A to E: Positive samples (amplicon size 206 bp), Lane F: Positive control, Lane G: Negative control, Lane H: 50 bp DNA marker.

**Figure-4:**
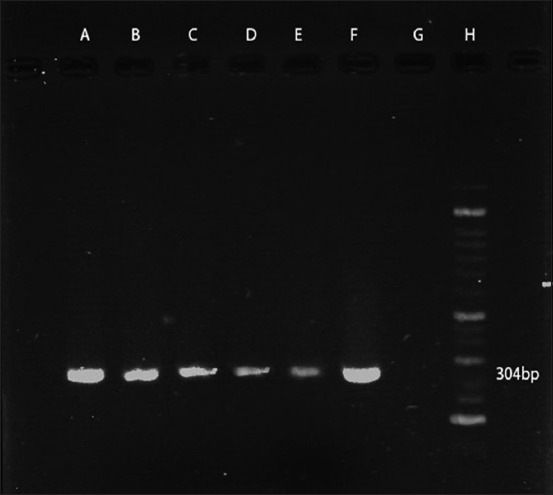
PCR amplification profile of *M. haemolytica* from DNA isolated directly from samples with 16s primer. Lanes A to E: Positive samples (amplicon size 304bp), Lane F: Positive control, Lane G: Negative control, Lane H: 50 bp DNA marker.

According to PCR test, using the stated primers, all the 38 isolates tested for the presence of *M. haemolytica* were identified as 100% positive (Figures-2-4).

**Figure-5:**
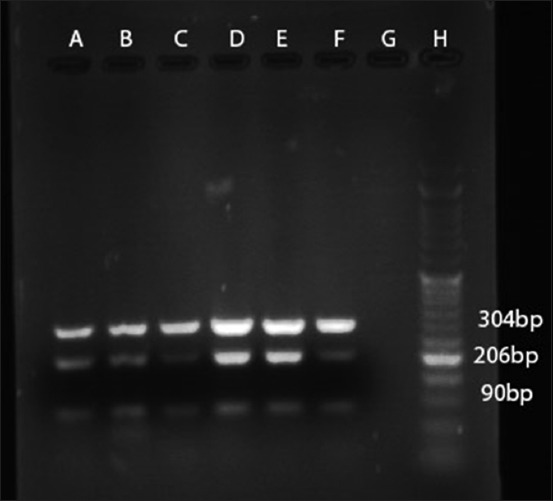
mPCR amplification profile of *M. haemolytica* from DNA isolated directly from samples. Lanes A to E: Positive samples (amplicon sizes 90 bp, 206 bp, and 304 bp), Lane F: positive control, Lane G: Negative control, Lane H: 50 bp DNA marker.

All the *M. haemolytica* were also positive with mPCR (Figure-5).

**Figure-6:**
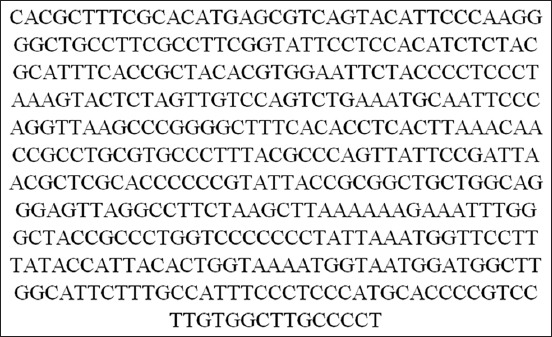
16S rRNA gene sequencing results of sample 55.

**Figure-7:**
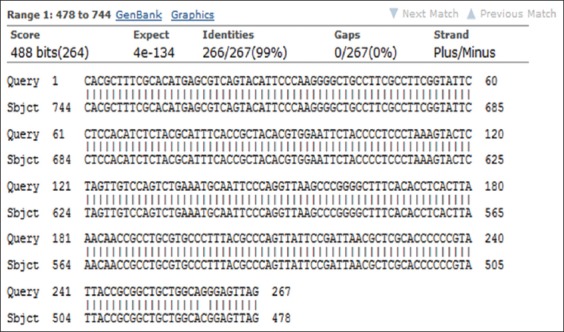
16S rRNA gene sequencing of *M. haemolytica* documented in NCBI database.

It should be noted that four isolates were shown 100% homology with the documented genomic sequencing of *M. haemolytica* in NCBI database (Figures-6 and 7).

## Discussion

Sheep and goat pneumonia is a syndrome that is caused by the reaction between factors of environmental stress, microorganisms, and host defensive ability. Pneumonia occurs in all ages of sheep and goats, in all breeds, and in every country of the world [6]. The high incidence of disease in warm regions and exhibition of a positive correlation with the area, suggesting the climatic condition, play a role in respiratory problems in that area. Factors such as crowding, dust, damp humid weather, or stress all can increase the disease [7]. Mortality caused by this disease has produced a serious economic loss and it can also lead to an enormous loss in the livestock industry. According to the reports of the veterinary organization in the project of sheep and goat pasteurellosis, Fars province was recognized as one of the most susceptible provinces to this disease. Considering the role of *M. haemolytica* in sheep pneumonia, identification and isolation of this bacterium can be effective in preventing this disease.

In general, many studies have been carried out regarding the isolation and identification of *M. haemolytica* using different methods [8-12].

As a result, in the present study, 38 *M. haemolytica* were isolated from 161 samples through culture and biochemical experiments that were finally confirmed through PCR test. Although results of PCR using specified primers provided the sufficient decimation for identifying the bacterium, mPCR was also utilized.

Abera *et al*. conducted a similar study in Ethiopia. They investigated 329 samples (185 nasal swabs and 144 lung tissues from abattoir) to identify *Pasteurella*. Among the 329 samples examined through PCR and culture, 14 samples (46.4%) were identified as *M. haemolytica* [10]. Results of our study support these findings, that is, all the 38 samples examined by PCR were positive for the presence of *M. haemolytica*. It should be noted that in our study, in addition to culture and PCR, mPCR was also utilized for more accurate identification and determination.

Findings of the current study were in line with the findings of a study by Kumar *et al*. in India. In their study, 12 isolates with culture and phenotypic characteristics of *M. haemolytica* were isolated, all of which according to PCR test were positive as *M. haemolytica* [13].

In another study carried out in the northeast region of Ethiopia aimed at identifying and isolating *Pasteurella* species through culture and biochemical experiments and determining the prevalence of them in pneumonia risk factors in sheep and goats of 988 nasal swabs and blood samples, 322 samples were pneumonic, while 79.5% of isolates were *M. haemolytica* [8].

Still, in another study conducted in Ethiopia, sheep pneumonic pasteurellosis was examined. A total of 256 samples were collected. *Pasteurella* and *M. haemolytica* were isolated from 64 samples (38 [59.4%] lung tissues and 26 [40.6%] nasal swabs). It is worth noting that all samples isolated from lung tissues were *M. haemolytica*, while 69% of nasal samples were *M. haemolytica*, which is strongly in line with our findings. Based on the findings of that study and the present study, it can be concluded that *M. haemolytica* is the main factor of pneumonic pasteurellosis in sheep [9].

Moreover, in a similar study by Alexander *et al.*, using the same primers as in our study, an mPCR was run and 15 previously isolated bacteria and 44 new isolates studied in their study were found to be positive as *M. haemolytica* [5].

Our findings showed that culture and PCR could be used for detection of *M. haemolytica*, but culture is very time-consuming, and PCR is very useful for fast identification of infectious agents. In addition, findings of the present study agree with the findings of Sabiel *et al*. who indicated that PCR is very useful and time saving for the identification of *P. multocida* and *M. haemolytica* [14].

## Conclusion

The present study indicated that culture and PCR are both practical in identification and isolation of this bacterium though culture is more time-consuming. The utilized mPCR has been more successful in the identification of the bacteria since it requires less time and cost. Furthermore, we propose the PCR as a superior method among other methods of bacteriology for fast examination of infectious diseases and mPCR, which is a valuable tool for identification of *M*. *haemolytica* in clinical samples of animals.

## Authors’ Contributions

This study is the major component of the work toward the M.Sc. thesis of FA. MT as supervisor of this work provided guidance during the entire experiment. FA wrote the first draft of the manuscript. Then, the whole manuscript was critically revised by MT. All authors have read and approved the final version of the manuscript.

## References

[1] Bojesen A.M, Larsen J, Pedersen A.G, Mörner T, Mattson R, Bisgaard M (2007). Identification of a novel*Mannheimia granulomatis*lineage from the lesions in roe deer (*Capreolus capreolus*). J. Wildlife. Dis.

[2] Angen Ø, Mutters R, Caugant D.A, Olsen J.E, Bisgaard M (1999). Taxonomic relationships of the*Pasteurella haemolytica*complex as evaluated by DNA-DNA hybridizations and 16S rRNA sequencing with proposal of*Mannheimia haemolytica*gen. nov, comb, nov*Mannheimia granulomatis*comb. nov*Mannheimia glucosida*sp. nov*Mannheimia ruminalis*sp. nov*Mannheimia varigena*sp. nov. Int. J. Syst. Evol. Microbiol.

[3] Zecchinon L, Fett T, Desmecht D (2005). How*Mannheimia haemolytica*defeats host defense through a kiss of death mechanism. Vet. Res.

[4] Quinn P.J, Markey B.K, Leonard F.C, Hartigan P, Fanning S, Patrick E.S.F (2011). Veterinary Microbiology and Microbial Disease.

[5] Alexander T.W, Shaun R.C, Yanke L.J, Booker C.W, Morley P.S, Read R.R, Gowe S.H.P, McAllister T.A (2008). A multiplex polymerase chain reaction assay for the identification of*Mannheimia haemolytica**Mannheimia glucosida*and*Mannheimia ruminalis*. Vet. Microbiol.

[6] Ozbey G, Muz A (2006). Isolation of aerobic bacteria from the lungs of chickens showing respiratory disorders and confirmation of*Pasteurella multocida*by polymerase chain reaction (PCR). Vet. Archiv.

[7] Weiser G.C, DeLong W.J, Paz J.L (2003). Characterization of*Pasteurella multocida*associated with pneumonia in bighorn sheep. J. Wildlife. Dis.

[8] Engdaw T.A, Alemneh A.T (2015). Pasteurellosis in small ruminants:Biochemical isolation, characterization and prevalence determination in relation to associated risk factors in Fogera Woreda, North-West Ethiopia. Adv. Biol. Res.

[9] Marru H.D, Anijajo T.T, Hassen A.A (2013). A study on ovine pneumonic pasteurellosis:Isolation and identification of*Pasteurella*and their antibiogram susceptibility pattern in Haramaya District, Eastern Hararghe, Ethiopia. BMC. Vet. Res.

[10] Abera D, Sisay T, Birhanu T (2014). Isolation and identification of*Mannheimia*and*Pasteurella*species from pneumonic and apparently healthy cattle and their antibiogram susceptibility pattern in Bedelle District, Western Ethiopia. Afr. J. Bacteriol. Res.

[11] Kaoud H, El-Dahshan A.R, Zaki M.M, Nasr S.A (2010). Occurrence of*Mannheimia haemolytica*and*Pasteurella trehalosi*among ruminants in Egypt. New York. Sci. J.

[12] Deressa A, Asfaw Y, Lubke B, Kyule M.W, Tefera G, Zessin K.H (2010). Molecular detection of*Pasteurella multocida*and*Mannheimia haemolytica*in sheep respiratory infections in Ethiopia. Int. J. Appl. Res. Vet. M.

[13] Kumar J, Dixit S.K, Kumar R (2015). Rapid detection of*Mannheimia haemolytica*in lung tissues of sheep and from bacterial culture. Vet. World.

[14] Sabiel Y.A, Musa M.T, Ann V.Z (2012). Identification of*Mannheimia haemolytica*and*Pasteurella multocida*by polymerase chain reaction and random amplification of polymorphic DNA. Sudan J. Vet. Res.

